# Navigating vaccination in pregnancy: Qualitative study in 21 ethnically diverse pregnant women

**DOI:** 10.1371/journal.pone.0310823

**Published:** 2025-01-31

**Authors:** Mohammad S. Razai, Michael Ussher, Lucy Goldsmith, Sally Hargreaves, Pippa Oakeshott

**Affiliations:** 1 Primary Care Unit, Department of Public Health and Primary Care, University of Cambridge, Cambridge, United Kingdom; 2 St George’s School of Health and Medical Sciences, Population Health Research Institute, City St George’s, University of London, London, United Kingdom; 3 Institute of Social Marketing and Health, University of Stirling, Stirling, United Kingdom; 4 The Health Foundation, London, United Kingdom; 5 Department of Health Services Research and Management, School of Health & Psychological Sciences, City St George’s, University of London, London, United Kingdom; 6 The Migrant Health Research Group, Institute for Infection and Immunity, City St George’s, University of London, London, United Kingdom; University of Surrey, UNITED KINGDOM OF GREAT BRITAIN AND NORTHERN IRELAND

## Abstract

**Background:**

Vaccination during pregnancy is crucial for safeguarding maternal and neonatal health, but vaccination rates remain suboptimal, especially in women from Black and Asian ethnic minorities. We explored the perspectives and decision-making processes of pregnant women regarding uptake of the three recommended vaccines in pregnancy: Influenza, Pertussis (whooping cough) and COVID-19. We also explored women’s attitudes to taking part in vaccine trials during pregnancy and the use of artificial intelligence (AI) to obtain information on vaccines.

**Methods:**

In 2023, we conducted in-depth telephone interviews with ethnically diverse pregnant women in the Greater London area using convenience and snowball sampling. The interviews focused on participants’ views on vaccination during pregnancy, participation in vaccine trials, information-seeking behaviours, and attitudes to emerging technologies for health information. Interviews were transcribed verbatim and thematically analysed. The data collection and analysis were conducted alongside the iterative development of the topic guide and coding framework, with key themes emerging through collaborative team discussions.

**Results:**

Twenty one pregnant women aged 20–39 were interviewed of whom 67% were from ethnic minorities and 29% were from migrant backgrounds. Half the participants (53%) reported hesitancy towards at least one of the vaccines. The analysis revealed several themes: concerns about vaccine safety, particularly regarding newer vaccines due to lack of long-term data; reliance on healthcare professionals for guidance, balanced with personal research; and a strong desire for clear and comprehensive information specifically tailored to pregnant women. Pregnant women reported insufficient information, explanation, or recommendation by midwives. Additionally, there was widespread refusal regarding participation in vaccine trials; and mixed responses to the use of AI (such as chatbots) for obtaining vaccine information.

**Conclusions:**

Pregnant women’s vaccination decisions are complex and require clear, unambiguous communication from healthcare providers, especially midwives, to address their specific concerns. Although information obtained via AI can be useful, responses were mixed.

## Introduction

Vaccination during pregnancy is crucial, protecting against preventable diseases (e.g., COVID-19, pertussis/whooping cough, influenza) that can compromise the health of both mother and child [1–3]. The influenza and COVID-19 vaccines protect the mother and newborn against severe disease, hospitalisation, complications, and death [1, 4]. In contrast, the pertussis vaccine mainly safeguards the infant through the passive transfer of immunity during pregnancy [5].

However, uptake of pregnancy vaccinations remains suboptimal in many countries, especially among ethnic minorities [6–8]. In 2021, confusion around COVID-19 vaccination during pregnancy is likely to have contributed to the deaths of 27 unvaccinated or partially vaccinated pregnant women in the UK [9, 10]. Data published in 2023, along with other MBRRACE-UK reports (Mothers and Babies: Reducing Risk through Audits and Confidential Enquiries across the UK), reveal significant and persistent disparities in maternal health. These disparities have had a detrimental impact on women from Black, Asian, and disadvantaged backgrounds [11, 12].

Between 2019 and 2021, infection with COVID-19 emerged as the leading cause of maternal deaths in the UK. A closer look at the demographics shows that these women were predominantly from ethnic minority backgrounds. Out of the 33 women who succumbed to COVID-19 during pregnancy or within six weeks postpartum, 14 were Asian, and five were Black [10]. Such disparities often arise from a complex interplay of cultural, systemic, and personal factors that act as barriers to vaccination [13–15]. Our recent systematic review showed barriers to vaccine uptake encompass concerns about safety, efficacy, and potential adverse effects, along with insufficient knowledge about benefits and perceived low disease severity. Facilitators include recommendations from trusted healthcare providers, easy access, clear communication on benefits and safety, and positive social influences [7]. Our recent meta-analyses showed a modest impact of interventions to boost influenza vaccine uptake [6]. We also examined patient, provider and policy level interventions. In observational studies, patient-level interventions, such as clear recommendations from healthcare professionals with reminders and tailored face-to-face interactions, effectively increased vaccine uptake. Provider-level interventions focused on educating professionals about vaccine safety and regular reminders to offer vaccinations. Policy-level interventions involved financial incentives, mandatory vaccination data in electronic health records, and ensuring vaccine availability [6].

While existing literature offers some insights into general barriers and facilitators for recommended vaccinations (COVID-19/pertussis/influenza) in pregnancy [7, 16], further research is needed to unravel the complexity, context and nuance of factors affecting vaccination decisions during pregnancy among ethnically diverse populations. Furthermore, no recent data exist on women’s perspectives about participating in vaccine trials or how they view artificial intelligence-based technologies for vaccine decision-making.

We conducted in-depth telephone interviews with ethnically diverse pregnant women in London, England. Our aims were to:

1. Identify the barriers and facilitators affecting vaccination decisions.
2. Understand attitudes towards participation in vaccine trials.
3. Explore the role of technology-enabled solutions, particularly AI, in informing these decisions.

London was chosen as the site of the qualitative study as pregnant women in London, particularly from minority ethnic groups, have notably low vaccination rates. In 2021–2022, maternal influenza vaccine uptake among Black Caribbean women in London was 11%, compared to the London average of 30% and the national target of 75% [17]. Similarly, maternal pertussis vaccine coverage in London decreased from 57% in 2019 to 39% in 2022 and 36% in 2023 [18]. COVID-19 vaccine uptake has also been lower in London, especially in socio-economically deprived areas and among minority ethnic groups [19].

## Methods

### Study design

We conducted semi-structured qualitative interviews by telephone to explore the experiences and perspectives of ethnically diverse pregnant women regarding vaccination during pregnancy. Qualitative methods are well-suited for analysing complex and nuanced issues, such as health-related decision-making, deeply embedded in sociocultural contexts [20]. The ethnic categories used in this study are self-reported and based on the UK census groups. This categorisation aligns with how patients are asked to identify their ethnicity when registering with a general practice. The interviews were conducted by a male researcher who self-identifies as Asian (MSR). We selected participants through convenience sampling from two practices and supplemented this with snowball sampling. Convenience sampling allowed us to quickly access diverse participants with relevant experiences. Convenience sampling was also chosen for its practicality and efficiency in gathering data, particularly given the time-sensitive nature of eligibility for seasonal vaccines and the gestational window (16–32 weeks) for receiving the pertussis vaccine. Snowball sampling was used to reach some individuals with sceptical perspectives on vaccination and AI-driven interventions, who were not easily accessible through conventional sampling methods. This approach suited the exploratory nature of our research, enabling us to gather rich insights.

We followed the Consolidated Criteria for Reporting Qualitative Research (COREQ) (S1 Checklist) [21].

### Participant recruitment

Potential participants were selected through a systematic search of two general practices’ electronic systems or when consulting with a General Practitioner (GP). Eligibility criteria included being currently pregnant and at least 18 years of age. We continuously monitored patient demographics to ensure a diverse sample in terms of age and ethnicity. Patients were contacted by a clinician from their registered practice and received a concise briefing about the study. Interested individuals were sent a participant information sheet and consent form via email, text, or post and invited to a phone interview at a convenient time. Most patients were recruited from the two GP practices in south London. We also used snowball sampling of other pregnant women introduced to us by participants. The women resided in inner-city, suburban, but not rural areas. We translated the consent form and information sheet in Arabic, Bengali, Pakistani Punjabi, and Urdu to accommodate language needs. Informed written consent was obtained from all participants, including consent for audio recording, by a clinician (MSR and PO) before their inclusion in the study.

### Ethics and informed consent

Ethical approval was granted by the Health Research Authority and Health and Care Research Wales (REC 22/WA/0091). Participants were assured that their involvement or refusal to be involved would not affect their clinical care. Each participant received a £15 gift voucher. Confidentiality and anonymity were maintained by assigning unique identification numbers to participants, securely storing all data, and anonymising quotes [22]. All potential participants were clearly informed that their involvement was voluntary and that they could withdraw at any time without losing the incentive. The £15 gift voucher was offered as a token of appreciation for their time rather than as compensation, and we ensured that its value was modest to avoid undue influence. Furthermore, six pregnant women declined to participate, and the data collected reflects a diverse range of opinions on vaccination, including half who were vaccine-hesitant, indicating that the incentive may not have biased the sample.

### Data collection

An interview topic guide was developed based on a review of the literature and expert consultations (S1 File). Topics included barriers and facilitators to vaccination [6, 7, 14], attitudes towards vaccine trials, and the role of technology in health decision-making. The topic guide was also informed by the Capability, Opportunity, Motivation-Behaviour (COM-B) model, ensuring that the questions addressed the behavioural determinants relevant to vaccination uptake [23]. Initially piloted with three pregnant women, the topic guide underwent further iterations.

Data were collected through semi-structured telephone interviews, which effectively gathered in-depth qualitative data while accommodating participants’ schedules and geographical locations [24]. Interpreters were offered to all participants in their preferred language, but this service was not required. Participants provided demographic information, including age, gestation, comorbidities, geographical location, country of birth, and education level. They were also asked if they would accept influenza, pertussis, and COVID-19 vaccines if offered at the time of the interview. Vaccine hesitancy was defined as the delay or refusal of any of the three recommended vaccines during pregnancy. Data collection and analysis were conducted concurrently [25]. and guided additional areas of questioning until thematic data saturation was achieved (a point where no new themes emerged, as agreed by all authors [26]). Despite the ethnic diversity of the participants, after about two-thirds of the interviews were conducted, the same themes began to repeat, with no significant new themes emerging. This observation was confirmed by the research team, leading to the decision to stop further recruitment. Regular discussions were held to assess theme consistency, and data collection continued beyond the point of saturation to verify the stability of the thematic structure.

### Data analysis

Audio recordings of interviews were anonymised, transcribed verbatim by a professional transcription service and checked for accuracy. Transcripts were analysed both inductively by identifying themes emerging from the interviews and deductively by focusing on topics outlined in the interview guide for thematic analysis [27]. After conducting the first ten interviews, the lead researcher (MSR) immersed himself in the data by repeatedly reading the transcripts. This approach was adopted to develop a nuanced understanding of the participants’ experiences and perspectives [28]. Initial codes were generated by MSR, followed by identifying themes through an iterative process [27]. To ensure the validity and reliability of our findings, we employed member checking [29]. We listened to a random sample of audio recordings to review the accuracy and validity of the transcripts. Key issues, concepts, and themes emerging from the data were independently identified and coded by MSR and PO. These initial codes were then debated and refined, leading to the development of an initial coding framework. This framework was subsequently discussed with the wider research team and further developed through a process of negotiated consensus. Sending transcripts to participants for comments and feedback was not feasible.

## Results

We conducted 21 interviews between September and November 2023 in London, UK, achieving a response rate of 78% (21/27). Interviews ranged from 12 to 33 minutes in duration. Around half (10/21) of participants identified as either Black/Black-British or Asian/British Asian, and one as mixed ethnicity (Table 1). The majority (15/21) were born in England. Regarding pregnancy, most (18/21) women reported no complications; 12 were in their third trimester, and 11 were nulliparous. The mean age of participants was 32 years, and most held university-level qualifications. Of those interviewed, half (11/21) either declined one of the three recommended vaccines, particularly COVID-19 or expressed uncertainty about receiving the vaccines. Among six women who declined participation in the study, lack of time was the most common reason.

**Table 1:** Demographic and clinical characteristics of 21 pregnant women interviewed.

Characteristics	Value
Age in years, mean (range)	32.0 (20–39)
Ethnicity	
Black African/Caribbean/Black British	4 (19%)
White British	7 (33%)
White Other	3 (14%)
Asian/Asian British Pakistani	5 (24%)
Asian/Asian British Indian	1 (5%)
Mixed	1 (5%)
Country of birth	
England	15 (71%)
Abroad	6 (29%)
Qualifications	
University graduate	12 (57%)
Secondary school	9 (43%)
Complications in pregnancy	
Yes	3 (14%)
No	18 (86%)
Comorbidities	
Yes	6 (29%)
No	15 (71%)
Gestation	
First Trimester	3 (14%)
Second Trimester	6 (29%)
Third Trimester	12 (57%)
Parity	
0	11 (53%)
1	7 (33%)
2	3 (14%)
Vaccine hesitant	
Yes	11 (53%)
No	10 (47%)
Numbers of vaccines received	
0	5 (24%)
1	7 (33%)
2	6 (29%)
3	3 (14%)

### Key themes influencing vaccination decisions in pregnancy

The thematic analysis of the 21 interviews with pregnant women revealed a range of themes regarding their perspectives on vaccination during pregnancy.

- Vaccine safety concerns
- Previous vaccination and illness experiences
- Trust in healthcare professionals and science
- Information-seeking and decision-making
- Role of family and social networks in decision-making
- Need for clear and comprehensive information
- Lack of information from midwives
- Views on participation in vaccine trials
- Attitude to AI-enabled technology for information

The following includes a description of each theme and relevant quotes.

### Vaccine safety concerns

A prevailing theme across the interviews was concern about the safety of vaccines during pregnancy, especially potential risks to the unborn child and the unknown long-term effects of newly developed vaccines. This anxiety was heightened due to the perceived lack of thorough research or long-term data, particularly regarding the COVID-19 vaccine. Participants’ reluctance often arose from their protective instinct towards their unborn child.

*" With the COVID vaccine, no, I’m not entirely sure about that. Just because I wouldn’t want to do anything that’s going to put my child at risk. P3 Black African*

*"I’m just not sure about the safety of the COVID vaccine for me and my baby. It’s all so new, and there’s a lot we don’t know yet." P17 British Indian*

*“I don’t feel very comfortable with vaccinations during pregnancy*, *to be honest*. *Just out of fear*, *really*, *about side effects*. *From what I’ve read and heard*, *pages on Google*. *I don’t remember the exact sources*, *not a lot of research is done in pregnancy women with vaccinations*. *So*, *it just concerns me about the long-term effects on baby*.*” P10 White British*

There was a widespread hesitancy towards recently developed COVID-19 vaccines, with a preference for those that have been extensively tested and established. This hesitancy was often rooted in concerns about the rapid development and approval processes and a lack of long-term data on the effects during pregnancy. This theme suggests the participants’ cautious approach towards newer vaccines, reflecting a broader scepticism and the need for clear and transparent information.

*"I would probably defer taking these very new [vaccines]… Something that was just developed recently I’d probably defer that until after the pregnancy. Because* [at] *the pilot stage or trial stage…the possible side effects or whether there are side effects or not, the possible effects on the mother and the child are not necessarily known extensively" P3 Black African*

*“Only because of the side effects and because it [COVID Vaccine] hasn’t been out as long as other vaccinations, so it makes me a bit wary of taking it.” P15 British Pakistani*

*"I just am a bit more hesitant because of the lack of research out there on it." P21 White British*

*"The COVID vaccine*, *I do have my reservations about*, *probably while I’m pregnant*. *I don’t have enough information on it " P18 British Pakistani*

### Previous vaccination and illness experiences

Participants reflected on how their own health conditions and past reactions to vaccines shaped their attitudes towards getting vaccinated during pregnancy.

*"I remember not reacting very well to the booster jab [COVID-19] at the time… I had very sore arms and I had a fever… so I just didn’t want to have to go through that in my current state [pregnancy]." P18 British Pakistani*

*“Oh, with the COVID, I did it because I have asthma and I had the COVID myself, so I went to take it…the aspect was more of saving my life than the negative aspect, so that’s why I went to take it.” P5 Black African*

*"I’ve had… I can’t remember now*, *three or four COVID vaccines*. *I’ve had COVID [infection] twice*. *The second time I had it was actually in March of this year*, *which was after a third or fourth jab*. *And have to say*, *I was very unwell*. *I haven’t had that length of time off work*, *I don’t think ever*.*”* P21 White British

### Trust in healthcare professionals and science

Trust in healthcare professionals and science was a significant factor influencing the women’s decision-making regarding vaccinations. This trust is built on past encounters with healthcare professionals and the expertise and experience of healthcare providers, with many participants indicating that they rely heavily on medical advice when deciding whether to get vaccinated. However, this trust is not absolute and can be challenged by personal beliefs or concerns, especially regarding newer vaccines. This reflects a balance between respecting medical expertise and maintaining personal responsibility in healthcare decisions. It also highlights the struggle between intuition and professional guidance, especially in the context of pregnancy, where emotional and physical changes are significant.

*“I guess I just trust my doctor. I just trust the NHS. Whatever information they have, I’m sure it’s for my best interest and for my health. I’ve never had a bad experience having a flu jab or a COVID jab, and I believe in the science backing it. And I just trust in the expertise of the professionals, rather than hearsay, or stuff in the media, or stuff that’s said on social media, as in conspiracy theories.” P7 British Pakistani*

*“I take my doctor’s advice, my doctor’s advice is to get the flu jab so I will be getting it whilst I’m pregnant, I haven’t heard anything about getting another COVID vaccine. If I get asked, I’ll have to think about it and do a bit more research.” P20 White British*

*"My decision is also influenced by my own beliefs about natural immunity*… *I think it’s important to consider all factors*, *not just what the doctors say*.*" P11*

### Information-seeking and decision-making

Many participants actively sought information from various sources, including scientific studies, healthcare providers, and personal networks, to make informed decisions about vaccines. This was driven by a desire to make informed decisions that are best for both the mother and the unborn child. It reflects a trend towards empowered healthcare choices, where patients play an active role in understanding the implications of medical interventions.

*"Before I make any decision about vaccines, I usually talk to my doctor and look up scientific studies." P2 White Australian*

*"I listened to a Radio 4 programme…they were talking to some doctors about the statistics of women who were having early births due to getting COVID… Then obviously the advice of my midwife and my GP was another thing. It’s hard not to be influenced by the media but of course you do get certain stories that stick with you and influence you. On the other hand, my peer group, a lot of my friends are pregnant and that kind of age group, so I guess it’s a discussion amongst friends. But yes, probably just asking for more information from the doctor and making a decision.” P20 White British*

*“Published medical journal-type things*. *So*, *that sort of thing*. *And there’s also the US medical website*. *There*, *we’d look*. *We wouldn’t just Google it and see what popped up*. *It would be reputable medical researcher places*, *so all the big important ones*, *I suppose*, *and mainly what the NHS advised as well*.*” P14 White British*

### Role of family and social networks in decision-making

The influence of family, friends, and social networks is a notable theme, indicating that decision-making around vaccines is often a collaborative process involving discussions with close ones, not just a personal decision.

*"My husband and I discussed it, and we also talked to the midwives. We were given quite a lot of information brochures as well by our hospital. It was a pretty straightforward one." P18 British Pakistani*

*"We looked at a lot, my husband looked at a lot of data and then ultimately it was my choice. When I went to see the GPs, they would obviously talk to me about it and the midwives." P14 White British*

*"When I was pregnant with my first daughter*, *I’d met a few mums through antenatal classes*, *and it would come up then*. *We’d discuss*. *I’d be like*, *have you had the vaccine or not*? *And just to see how they had felt about it*. *And it was very mixed*. *Some had*, *some hadn’t*. *But I think the ones that had maybe looked into it a little bit*, *which was quite interesting*.*” P19 Black Caribbean*

### Need for clear and comprehensive information

Participants emphasised the need for clearer and more thorough information about the risks and benefits of vaccines, especially for newer ones like COVID-19. They sought information tailored to pregnant women, highlighting the need for advice that addresses their unique health concerns. This theme reveals a gap in health communication strategies, emphasising the need for more targeted information dissemination to pregnant women.

*“A link to a video that is an awareness training, so to speak. So that people can understand what the vaccine does and why it’s important for them… Probably add experiences of a parent who is willing to talk about, okay, them having vaccines and how it helped them.” P3 Black African*

*"Just echoing what I’ve said before, I think maybe if that information is made available at a set appointment where it’s written that at week 18, for example, the appointment is when we go through these tests or a discussion. There’s also a chance to have a chat about which vaccines you can take." P18 British Pakistani*

*“I think wherever you are scheduled*, *your midwife*, *midwifery team*, *and really the people looking after you through your pregnancy*. *I think*, *particularly when it’s the first pregnancy*, *you end up putting a lot of trust in them*. *So I think if they can provide the information*, *whether that be in reading form of patient leaflets or links to research papers… So I think it’s the sort of subject that unless you’re well read on*, *it’s quite difficult to make that informed choice*, *which is why I think everyone takes the advice of the midwife or of the [NHS hospital] Trust that they’re registered at*.*” P21White British*

### Lack of information from midwives

Most participants mentioned insufficient information and guidance from midwives regarding vaccinations during pregnancy. Participants desired detailed, clear explanations to understand the necessity and timing of vaccines. The lack of communication appears to contribute to uncertainty and hesitancy about vaccination decisions. Specifically, pregnant women pointed to an over-reliance on written materials without personal engagement. Some also reflected on the ambiguity in professional advice about the COVID-19 vaccine, leading to confusion and caution among expectant mothers.

*"It’d be helpful if [midwives] gave us some stats about why we need this vaccine and how it helps." P7 British Pakistani*

*“I basically only ever got told that these are the vaccinations that I need to have during pregnancy. They never really explained why you would need them. So I suppose maybe people weren’t as well informed or didn’t go look into it themselves. They would maybe sometimes not get them because they don’t realise that they need them… I know with the midwives, they just pointed me to a QR code and said, “Read this”. But when I looked at it, it was just about whooping cough, what it does. So it did explain a little, but it didn’t really explain why you needed to have it in pregnancy.” P1 White Irish*

*“But I asked at my first midwife appointment… And they said that the flu and pertussis was recommended …not recommending COVID vaccine*. *And I thought that was quite interesting*. *The fact that they are not now saying that COVID’s necessarily a recommended one to have*. *They’re just remaining neutral*. *Which just made me a little bit hesitant as to*, *is this because there’s just not enough studies or are there concerns*?*” P21 White British*

### Views on participation in vaccine trials

Concerns about the ethics and safety of participating in vaccine trials during pregnancy were evident, particularly regarding the impact on the unborn child. The women were particularly wary of the potential risks to their unborn children and the ethical implications of involving the foetus in such trials. This theme reflects a broader concern about the perceived vulnerability of pregnant women in medical research and the need for careful consideration of the ethical dimensions of involving this population in trials. It highlights the need for rigorous ethical standards and informed consent processes in research involving pregnant women.

*"I would never participate in a trial while I was pregnant…I view it as unethical. I don’t think that ethically it’s my decision to test on both me and the unborn baby." P11 White other*

*"If it was a brand new vaccine that they wanted to try solely for pregnant women, I think I probably would be a little bit nervous about that." P16 White British*

*“I think a lot of women, personally, would be against it [vaccine trials] because no one wants to be the guinea pig and no one wants to make their child the guinea pig. That’s the thing. And there is still a lot of stigma from previous things that were given that have caused limb deformities and things like that.” P17 British Indian*

*"I wouldn’t volunteer to be tested for a vaccination whilst pregnant. It may be because I’m quite nervous to do so because I’ve had a miscarriage, and I’m very protective over my pregnancy. But I’m sure other people who haven’t experienced what I’ve experienced would be protective over their pregnancy as well. So I don’t think people would be keen to do that. " P7 British Pakistani*

*"Personally, I wouldn’t undertake a medical trial while I was pregnant. For me, it would just be too much of a gamble." P14 White British*

*“I would rather not take that risk*. *I’m happy to do that when it’s tried and tested*, *but I don’t want to be the guinea pig*.*” P19 Black Caribbean*

Solutions proposed to increase pregnant women’s participation in vaccine trials included providing balanced information about the vaccine’s benefits and risks, offering financial incentives, and ensuring clarity about the research’s expected outcomes. Additionally, evidence of a vaccine’s safety from trials in the general population could increase their confidence in participating. These are crucial in tackling the concerns and priorities of pregnant women considering participation in clinical trials.

*“So, it would depend on what the trial was. If it was for a well-established vaccine that we knew was generally safe, but it just hadn’t been tested in pregnancy, I’d probably be more susceptible to doing it.” P16 White British*

*“I suppose … seeing the good that it brings as well. Like having peoples’ experiences about it and that sort of stuff. Maybe having some professionals speak about the vaccine and tell us the benefits and everything like that. And that [benefits] outweigh the risks … Describe it for us, what the side effects could be, what are the benefits of it, what are the risks in taking it, which there always are. Which I feel like they were a bit… in COVID terms anyways, reluctant to mention the risks of taking a vaccine, which there always is with all vaccinations, and I understand that. But, just, yes, being balanced with the information would be good.” P1 White Irish*

*“Probably a financial incentive would get people to join. I don’t think people would volunteer for it openly. But I think, as there are medical trials that offer thousands of pounds for you to join depending on how risky it is, I think that would definitely bring people in. It wouldn’t bring me in, I would still be averse to it. But I just think that people that obviously are struggling..yes, a financial incentive for them.” P6 British Pakistani*

*“Clear information about the benefits*, *the risks*. *When you’re pregnant your focus is on the baby*, *so it’s going to be what are the risks and benefits to me as a parent but also what are the benefits and the risks to the baby*.*” P12 White British*

### Attitudes to AI-enabled technology for information

Some participants showed openness to using AI for obtaining vaccine information, suggesting a potential role for technological solutions in vaccine communication. However, they suggested its usefulness as an initial engagement tool and not a substitute for personal interaction with a healthcare professional.

*"Yes. I think that’s [chatbot] an ideal way of gaining more information. Definitely." P7 British Pakistani*

*“I feel like that [chatbot] would be a good idea to get a quicker response, and to get information. But sometimes just speaking to an actual person is better, because then the person that they speak to can give examples on different patients that have taken it, and their views on things. But the AI thing is also a good idea, I believe.” P4 Black African*

*“I would say as a preliminary discussion or intro to the vaccine*, *that [chatbot] could help… But I would prefer to*, *apart from the bot system*, *talk to someone with more knowledge in the medical industry*, *previous patients*, *or previous moms whose done it while they’re pregnant*.*” P3*, *Black African*

However, there was also scepticism about the accuracy and source of the information. Concerns were raised about whether the data is vetted by healthcare professionals or pulled from potentially unreliable internet sources. Additionally, the functionality of chatbots was criticised for being clunky and unresponsive. Preferences tended towards supplementing initial AI interactions with advice from medical experts and the experiences of others who have been vaccinated during pregnancy. Some participants had a strong aversion to chatbots with doubts about their capability to handle nuanced inquiries and influence significant health decisions, emphasizing the value placed on human interaction and expert opinion over automated responses.

*“Maybe a little bit sceptical, just wondering how the bot has the information. Where it came from, it was supplied by a professional … or if it’s pulling it from the internet. So from social media and sites and things like that. I would just wonder where it’s getting its information from… and whether it’s correct, the type of information, or if its just there being obviously supplied by another… As a purpose to get somebody’s opinion across rather than it being a scientific fact sort of thing.” P1 White Irish*

*“I probably would be less inclined to use something like [AI chatbot] because I find often, they’re clunky and not super useful or responsive.” P2 White Australian*

*“I hate those things [chatbots] with a passion. I find that they are not nuanced or responsive enough to give answers to that question…I don’t think those people would be convinced by an impersonal chatbot. I can’t be convinced by a chatbot to make a purchase online, let alone somebody who doesn’t have medical knowledge being convinced to go and get a vaccine while they’re pregnant.” P11 White*

*"If there was a chatbot*, *I think people might be wary of whether they want to put any personal information in due to data breaches*. *So the ramifications of that should be told to people using chatbots*.*" P7 British Pakistani*

## Discussion

### Principle findings

This study on the attitudes and decision-making processes of pregnant women towards vaccinations revealed several key themes, including concern for vaccine safety, particularly regarding newer vaccines such as COVID-19. Women expressed apprehension about the potential risks these vaccines pose to their unborn children, emphasising the need for proven safety and efficacy. The hesitancy was often rooted in the lack of long-term data and the rapid development of vaccines. Another significant finding is the trust placed in healthcare professionals. Despite this trust, many women still engaged in personal research, reflecting a desire to make informed decisions that are influenced, but not dictated, by medical advice. This blend of trust and autonomy in decision-making highlights the critical role of healthcare providers in offering guidance while respecting individual choices.

Additionally, the study found a lack of clarity and guidance from midwives, highlighting the need for more thorough and accessible information tailored to pregnant women. Many participants voiced their concerns regarding participation in vaccine trials during pregnancy, where the potential risks to the unborn child weighed heavily. Lastly, there was a mixture of views on using AI and technology to obtain vaccine information.

### Strengths and limitations

To our knowledge, this study is the first to explore the perspectives of pregnant women on all three recommended vaccines during pregnancy at the time of the study, along with their attitudes towards emerging technologies and vaccine trials. All participants were pregnant during the interviews, and there was a broad range of ages, vaccination status and educational backgrounds. Nearly half of the participants identified as Black African/Black Caribbean or British Asian/Asian Pakistani, groups who generally have lower vaccination rates than the White British population. The concurrent, iterative process of data collection and analysis, and in-depth interviews provided new insights. The cohort included women who were accepting of vaccines and those with vaccine hesitancy, ensuring a broad spectrum of opinions. Additionally, the mixed perspectives on using AI and technology for disseminating vaccine information during pregnancy represent relatively new dimensions in vaccine research and emerging trends in healthcare communication [30].

The study has several limitations. While convenience and snowball sampling provided valuable insights, we acknowledge the inherent limitations of these methods, particularly in terms of generalisability. The sample size and limited diversity mean that the findings may not represent the broader population but should be interpreted as reflective of the specific groups engaged in the study. Also, we did not fully explore cultural factors or language barriers to vaccine acceptance.

Another limitation is the risk of research bias. The research team engaged in critical reflexivity to examine how their personal and professional backgrounds, including ethnicity, gender, and socio-economic status, might have shaped the data collection, analysis, and interpretation processes. Recognising the potential impact of the team’s demographic composition, especially the ethnic mismatch between the researchers and participants from minority ethnic groups, the team actively considered how these differences might influence participant responses. This awareness was particularly pertinent given the potential for participants to adjust their responses based on perceived social hierarchies or professional dynamics [30].

The team had regular reflexive discussions to reduce this risk, allowing for continuous critical assessment of their positionality throughout the research process. These discussions aimed to identify any unconscious biases that might arise during interactions with participants and in the interpretation of data. Furthermore, to minimise the impact of the researchers’ professional status and clinical relationships, efforts were made to create a more neutral and supportive environment during interviews, encouraging participants to express their views freely. The potential influence of the interviewer’s gender was also considered, as it could affect how participants responded, particularly in contexts where gender dynamics are culturally significant. Moreover, the possibility of social desirability bias—where participants might provide responses they perceive as more socially acceptable to the interviewer—was acknowledged. To counteract this, the team employed strategies such as ensuring anonymity and confidentiality, clearly communicating that there were no right or wrong answers and emphasising the importance of honest responses.

Although efforts were made to include participants with limited English through interpreters and translated materials, language barriers might still have led to their underrepresentation, potentially introducing selection bias. Finally, as interviews were conducted at a single point in time by one researcher, changes in participants’ vaccination decision-making over time remain unexplored.

### Comparison with existing literature

Some of the findings of this study align well with existing literature on the topic [6–8]. Concerns about vaccine safety during pregnancy have been a recurring theme in qualitative research [8, 16]. The trust in healthcare providers and the pivotal role of midwives mirror findings from previous studies [6, 8, 14]. The hesitancy towards new vaccines, particularly the COVID-19 vaccine, is consistent with the global sentiments observed during the pandemic [13, 31]. Our participants’ views of vaccine trials align with qualitative data from some European countries collected before the COVID-19 pandemic, indicating that pregnant women strongly rejected participation in vaccine trials [32]. Historically, pregnant women have been underrepresented in or excluded from vaccine trials, resulting in a significant gap in understanding the effects of vaccines during pregnancy [33–35]. This omission hampers the development of evidence-based guidelines and raises ethical concerns, as the lack of data leaves pregnant individuals and their healthcare providers navigating a decision-making vacuum [33]. On the other hand, although vaccine development generally undergoes a rigorous safety process, pregnant women may be concerned about novel vaccines’ effects on foetal development and pregnancy [8]. In light of the COVID-19 pandemic, there have been calls to include pregnant women in vaccine trials [36].

As in our study, the role of midwives is crucial. A 2024 qualitative systematic review of 22 studies of midwives’ experiences in addressing health behaviour change, including vaccination, within routine maternity care revealed that discussions about health behaviour change are often sidelined in clinical practice [37]. Despite midwives acknowledging the significance of these conversations, they were not consistently prioritised. This finding suggests a gap in integrating health behaviour change dialogues within routine care. Therefore, any proposed intervention should focus on equipping midwives with the tools and skills necessary for effective health behaviour change communication to facilitate vaccine uptake during pregnancy. Additionally, systemic factors, including workforce shortages, funding limitations, and institutional policies, play a crucial role in either enabling or constraining midwives’ capacity to fully engage with their patients [31]. Consequently, while the role of midwives is vital, it must be considered alongside these broader systemic challenges.

Guidance on maternal vaccination may change. The US Centers for Disease Control and Prevention Advisory Committee on Immunization Practices recently recommended respiratory syncytial virus (RSV) vaccination for pregnant persons at 32–36 weeks’ gestation during the RSV season (September–January) to protect infants from RSV-associated lower respiratory tract infections, with a choice between maternal vaccination and infant receipt of nirsevimab [38, 39]. From September 2024, the UK government has recommended RSV vaccination during pregnancy starting at 28 weeks of gestation [40]. Vaccines targeting Group B Streptococcus are in development, with expectations of their release within the next five years, marking important strides in maternal and infant health protection [41, 42].

These recent studies show it is crucial to keep up to date with the evidence on vaccination in pregnancy. There is a critical need for clear, supportive communication from healthcare providers to address vaccine hesitancy and improve uptake, alongside awareness of the evolving landscape of vaccine recommendations.

Furthermore, in the era of digitalisation, technology-enabled solutions, particularly AI, have been increasingly harnessed for healthcare information dissemination and decision-making [43, 44]. AI has the potential to bridge the knowledge gap and overcome language or cultural barriers that might affect healthcare choices during pregnancy. The emergence of sophisticated chatbots powered by advanced natural language processing (NLP) technologies and large language models (LLMs), such as OpenAI’s ChatGPT, marks an important shift [45]. ChatGPT and similar platforms leverage deep learning algorithms to understand and generate human-like text, allowing for more meaningful and contextually relevant user interactions [46]. This technological advance could enable chatbots to offer personalised healthcare guidance for vaccine uptake with a level of precision previously unattainable in earlier models.

However, when discussing AI in relation to vaccination during pregnancy, the most important issues include addressing algorithmic biases that can exacerbate existing disparities. To avoid reinforcing systemic biases, it is crucial to ensure the representation and inclusion of diverse datasets. Additionally, transparency, safety, accuracy, and explainability of AI systems are essential, alongside continuous monitoring to evaluate AI’s impact on health.

Implementing AI technologies, such as chatbots, into clinical practice is challenging in primary care [47]. AI should be designed to augment information dissemination methods, such as face-to-face consultations, printed educational materials, and telephone advice services, rather than replace them. Additionally, stakeholders must earn the trust of GPs by ensuring that these technologies are evidence-based, improve patient care, and do not add to the workload of an already overstretched healthcare system.

## Conclusions

This study highlights the multifaceted nature of vaccination decision-making among this group of pregnant women. It contributes to a deeper understanding of the complex interplay of personal and systemic factors that shape vaccination choices. It offers healthcare providers, policymakers, and health technologists valuable insights into tailoring more effective interventions.

**Fig 1:**
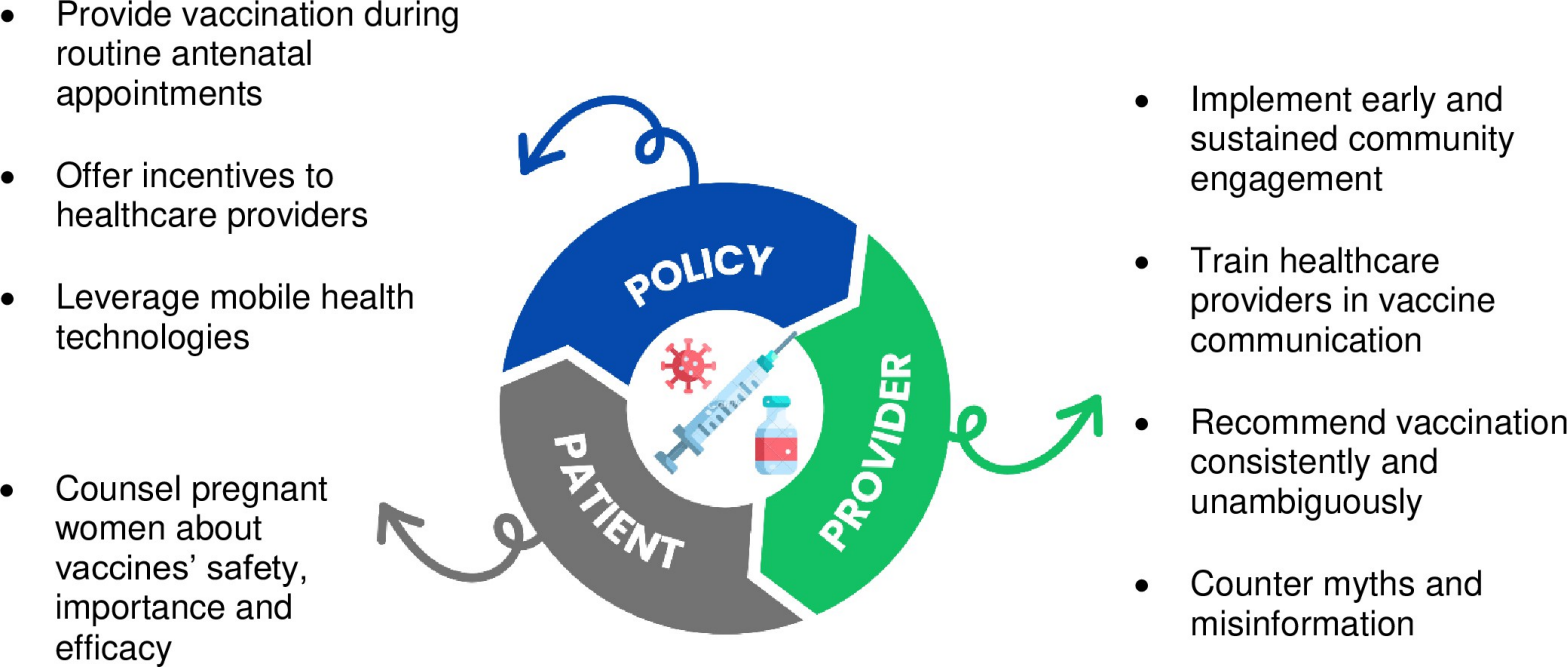
A multifaceted approach to increase vaccination in pregnancy: The ‘3 Ps’ (patient, provider, policy) (adapted from Razai et al. [6]).

Concerns regarding vaccine safety, especially for newer vaccines like COVID-19, underscore the need for transparent communication from healthcare providers. The balance between trusting medical advice and maintaining personal agency in decision-making indicates a need for a nuanced approach in healthcare interactions. **Fig 1** shows possible ways at the patient, provider, and policy levels, the ‘3 Ps’, to increase maternal vaccine uptake in diverse populations [6].

Healthcare providers should be aware of pregnant women’s concerns and address them accordingly. Additionally, the potential for AI and technology to aid in information dissemination in healthcare settings should be explored, leveraging these tools to enhance patient education and decision-making. Future research could include longitudinal studies to track changes in vaccine hesitancy over time and comparative studies across different regions or cultural contexts to explore local influences on maternal vaccination decisions.

## Supporting information

S1 Checklist COREQ (COnsolidated criteria for REporting Qualitative research) checklist. (PDF)

S1 File (DOCX)
