# Supplementary material for: Navigating vaccination in pregnancy: Qualitative study in 21 ethnically diverse pregnant women
Source: PLoS One. 2025 Jan 31;20(1):e0310823. doi: 10.1371/journal.pone.0310823 (PMC11785291; doi:10.1371/journal.pone.0310823)
Supplement: S1 File — (DOCX) [file pone.0310823.s002.docx]

**Rec Ref: 22/WA/0091 IRAS ID: 312878**

**Topic Guide**

**Study Title: Facilitators and Barriers to Vaccine Uptake in Ethnically Diverse Pregnant Women: A Qualitative Study**

**Introduction**Thank you so much for your time and for taking part in the research interview today. The research is based at St George’s University, and the interview today is to understand your perceptions of COVID/influenza/pertussis vaccines, in addition to your ideas, concerns and expectations. Your contribution will also help us better understand views on Covid-19 vaccination and can help plan vaccination programmes. Please be aware that we would just like to understand your views on the questions we ask, and **any views you express will not affect your care in any way**.

- Check they have received the information sheet.
- Check they have given consent.
- Check they have answered the demographics questions.

*Do you have any questions? Are you happy to go ahead?*

***Reiterate that there’s no right or wrong answer***

***Start Recording***

***Warm up discussion***

1. **How has the COVID pandemic affected you?**

*Prompts:*
- Impact on home life, work, emotional impact
- Impact on your pregnancy and relationship
- Emotional impact
- Knowledge of others affected or dying

***More detailed exploration***

1. **Different people have different beliefs about vaccination during pregnancy. Are you able to share a bit about your thoughts on it?**

*Probes:*
- Could you tell me, how you came to that belief/understanding? How common is this belief amongst people you know? Where do you think they came to that belief?
- Is this view shared widely among your family and friends? / Do you have family or friends that have views which are very different from yours?
- Where did you hear about the information on which you formed that opinion?
- Do you think you are at risk from the virus? What affects this?

*Prompts:*
- Some people believe that COVID/Influenza/Pertussis ***doesn’t actually exist / is a conspiracy / affects people from different backgrounds differently.*** What are your thoughts on that?

***Focusing into key areas***

1. **What are your views on vaccines in general? (highlight the must ask questions or put in a separate table)**

*Probes:*
- Have your views changed over time? / Has anything changed or affected your views?

- Has the pregnancy changed your views on vaccines? How has it changed your views? Why has it changed your views? Anything else?
- Is that your opinion on all vaccines or does it vary from vaccine to vaccine? Why?
- How did you come to that belief/understanding?

-Do you think that vaccines should be taken during pregnancy? How did you come to that belief/conclusion? Can you tell more? Anything else?

- Would you apply these views to the COVID vaccination as well?
- Is this view shared widely among your family and friends? / Do you have family or friends that have views which are
 very different from yours?

*Prompts:*
- What have you heard about vaccines in general? What have you heard about vaccines in pregnancy?
- Some people believe vaccines are not needed as ***there are other ways to protect yourself against viruses***. What
 are your thoughts on that?

1. **What have you heard about vaccines during pregnancy?**

*Probes:* **-** What is your opinion on that?
- Where did you hear that information/view?
- Do you think this information is reliable? / What is your opinion of the information given by ***{source cited}?***
- Is this view shared widely among your family and friends? / Do you have family or friends that have views which are
 very different from yours?

*Prompts:*- Have you heard any information about any vaccine that worries you?
- Some people get a lot of information about the vaccine from: ***the government and NHS /family and friends / social media groups and online / religious or community leaders.*** What are your thoughts on that?

1. **On your questionnaire, you said that you: *would take / would not take / are not sure if you would take* the COVID/influenza/pertussis vaccine if you were offered it at the moment. What factors did you consider in making that decision?**

*Probes:* **-** What is your understanding of what COVID/influenza/pertussis vaccine does/role? Why do you think it is being offered during pregnancy?
- How did you come to that belief/understanding?
- Is this view shared widely among your family and friends? / Do you have family or friends that have views which are very different from yours?
- Where did you hear about the information on which you formed that opinion?
- How does your faith or background affects your views on the vaccination, if at all? If so, how?

*General Prompts:*

- Do you think there are any benefits from having a vaccine during pregnancy? If so, what are they? How your views be different if you were not pregnant? Why?
- Do you think there are any harms from having a vaccine during pregnancy? If so, what are they?

*Specific Prompts:*
- Some people feel there are ***problems with the vaccines for different communities/groups during pregnancy.*** What are your thoughts on that?
- Some people are worried about the ***ingredients in the vaccines / research or process by which coronavirus vaccines have been made/ way the vaccines will be given.*** What are your thoughts on that? Would your views be different if you were not pregnant?

1. **How would you describe your experience of healthcare in general?**

*Probes:*
- Have you ever had any bad experiences?
- Do you use healthcare services a lot? If not, do you seek advice about your health from somewhere else?
- What is your relationship like with your GP / *other healthcare professional they mention?*- Has this changed during the pandemic or impacted upon your approach to the virus and vaccine?

*Prompts:*- Some people don’t believe their doctors **are giving them accurate information about the virus/vaccine**. What are your thoughts on that?
- Some people are worried about taking the vaccine because **they don’t believe the doctors have their best interests at heart.** What are your thoughts on that?

-Have you ever **'felt you were treated differently' / 'felt left till last' / 'received unfair treatment'** when receiving healthcare in the UK?

1. **We are interested in trying to understand what might encourage people to take the coronavirus/flu/whooping vaccines to protect themselves. Is there anything that *would change your mind and make you want to take the vaccine / you think would change the mind of those you know who are worried about the vaccine?***

*Probes:*- Why do you think that might change *your/their* mind?
- How do you think that would change *your/their* understanding or perception?
- Who do you think could change *your/their mind on this?*- Tell me a bit more about why that is important to *you/them*? *Prompts:*

***-***Do you think having COVID/flu/whooping vaccine offered to you by a healthcare professional like a GP or midwife or hospital doctor during your routine antenatal clinic change your mind?

- Do you think seeing someone like a community champion offer the vaccine change your mind?

- Do you think a role model or a family or friend take the vaccine makes a difference?
- Do you think **seeing celebrities or people from your community receive the vaccine** makes a difference? Why?
- Do you think receiving **more information about the vaccine** / **different information about the vaccine** /

**Information about the vaccine from a different source/different formats** would change *your/their* mind? Why?

- How do you think a healthcare professional like GP or midwife can help change the mind of those worried about the vaccines?
- How do you think people from your community or community leaders can help change the mind of those worried about the vaccines?

- How do you think offering in a diverse range of locations such as pharmacies, community centres, pop-up facilities in high streets or supermarkets makes a difference?

- How do you think getting regular reminders about taking vaccine make a difference?

**Participation in Vaccine Trials During Pregnancy**

1. Can you describe your thoughts or experiences regarding participation in vaccine trials during pregnancy?
   - **Probes**: What factors influenced your decision? How did discussions with family, friends, or healthcare providers play a role in your decision-making process? Where did you primarily get your information about the vaccine trials? Can you describe any emotional responses you had when considering participation in the trial?
   - **Prompts**: Consider aspects like safety, information provided, or advice from healthcare professionals. Think about any conflicting opinions or support you received. Consider how trustworthy and comprehensive this information was.

**Follow-up Question**: How did you perceive the risks and benefits associated with these vaccine trials?

- - **Probe**: Were there any specific concerns or assurances that stood out for you?
  - **Prompt**: Think about both short-term and long-term effects on you and your baby.

**Use of AI-Enabled Technologies (i.e Chatbots) During Pregnancy**

1. Have you used any AI-enabled technologies, like chatbots, for pregnancy-related information or support? If so, can you share your experience?
   - **Probes**: What kind of information or support were you seeking? How did you assess the reliability of the information provided by AI technologies? Did you feel that the AI technology was tailored to your specific needs and concerns? How does your experience with AI technology compare to interactions with healthcare professionals?
   - **Prompt**: Examples could include health advice, appointment scheduling, or emotional support. Consider any cross-checking with healthcare providers or other sources. Think about instances where the technology did or did not seem to understand your unique situation. Consider aspects like empathy, depth of interaction, and response time.

**Follow-up**: How did the use of these technologies affect your pregnancy experience?

- - **Probe**: Did you find these technologies reliable and helpful?
  - **Prompt**: Consider aspects like ease of use, accuracy of information, and level of emotional support.

**Additional**: In what ways do you think AI technologies could be improved to support pregnant individuals better?

- - **Probe**: Are there specific features or types of support you felt were missing?
  - **Prompt**: Think about personalisation, interaction quality, and information relevance.

**That’s everything I wanted to ask you. Is there anything else you would like to add or think I should have asked? Is there anything you would like to ask me?**

**Thank you very much for taking the time to give us your views. Your contribution to this research will help us to think how best to discuss vaccination during pregnancy.**
